# Animal Models of Multiple Myeloma Bone Disease

**DOI:** 10.3389/fgene.2021.640954

**Published:** 2021-06-07

**Authors:** Syed Hassan Mehdi, Sana Nafees, Syed Jafar Mehdi, Carol A. Morris, Ladan Mashouri, Donghoon Yoon

**Affiliations:** ^1^Myeloma Center, The University of Arkansas for Medical Sciences, Little Rock, AR, United States; ^2^Department of Biosciences, Jamia Millia Islamia, New Delhi, India

**Keywords:** mouse model, C57BL/KalwRij, 5TMM, *MYC* derived model, SCID-hu MODEL, SCID-rab MODEL, multiple myeloma bone disease, multiple myeloma xenograft model

## Abstract

Multiple myeloma (MM) is a clonal B-cell disorder characterized by the proliferation of malignant plasma cells (PCs) in the bone marrow, the presence of monoclonal serum immunoglobulin, and osteolytic lesions. It is the second most common hematological malignancy and considered an incurable disease despite significant treatment improvements. MM bone disease (MMBD) is defined as the presence of one or more osteolytic bone lesions or diffused osteoporosis with compression fracture attributable to the underlying clonal PC disorder. MMBD causes severe morbidity and increases mortality. Cumulative evidence shows that the interaction of MM cells and bone microenvironment plays a significant role in MM progression, suggesting that these interactions may be good targets for therapy. MM animal models have been developed and studied in various aspects of MM tumorigenesis. In particular, MMBD has been studied in various models, and each model has unique features. As the general features of MM animal models have been reviewed elsewhere, the current review will focus on the features of MMBD animal models.

## Introduction

Multiple myeloma (MM) is a plasma cell (PC) malignancy that represents an accumulation of terminally differentiated monoclonal PC in the bone marrow (BM) (Katz, 2010). It is the second most hematological threat with a new case rate, 7.0 per 100,000/year based on 2013–2017 cases in the United States (National Cancer Institute: Surveillance, Epidemiology, and End Results Program, 2020). In 2020, it was estimated as 32,270 cases of MM (1.8% of all new cancer cases) and 12,830 deaths (2.1% of all cancer deaths) in the United States (National Cancer Institute: Surveillance, Epidemiology, and End Results Program, 2020). In almost all cases, MM is preceded by a premalignant disease known as monoclonal gammopathy of undetermined significance (MGUS) (Weiss et al., 2009; Landgren et al., 2019). During the progression of MGUS to MM, not only that complex genetic changes occur in the PCs, but also changes in the BM microenvironment (BME) occur, including angiogenesis, immune suppression, and increasing bone resorption (Kyle and Rajkumar, 2004). MM bone disease (MMBD) is defined as the presence of one or more osteolytic bone lesions or diffused osteoporosis with compression fracture attributable to the underlying clonal PC disorder (Rajkumar et al., 2014). MM is a paradigm of tumor–stroma interdependence, where MM cells establish tight contacts with the stroma. The osteoclast (OC) activity increases in areas adjacent to myeloma cells, whereas the osteoblast (OB) activity declines (Bjorkstrand et al., 2011). MMBD is found in 79% of patients with newly diagnosed MM patients (Kyle et al., 2003). A substantial improvement in overall survival was made in the past few decades. However, a significant number of patients still develop osteolytic lesions, marking a major pitfall under the current management (Silbermann and Roodman, 2013; Kumar et al., 2014; Ring et al., 2018). The lytic lesion is highly associated with MM’s devastating outcomes, including pathological fractures, debilitating pain, and increased mortality risk (Saad et al., 2007). An assessment of fracture effect on survival after MM diagnosis in the large Sweden population revealed that a fracture significantly reduced a survival risk (Thorsteinsdottir et al., 2020). Furthermore, MMBD often results in reducing patient’s quality of life and survival via hypercalcemia and spinal cord compression syndromes (Marino and Roodman, 2018).

MM therapy has significantly improved by an increased understanding of mechanisms that promote the disease’s onset and progression (Botta et al., 2017). Myeloma animal models contribute significantly by reproducing the pathological features and providing insight into the interactions between MM cells and the BME. In addition, these models serve as a tool to investigate and predict the effectiveness of novel therapeutic strategies that can translate into the clinic. Despite the significant advance in the understanding of disease pathophysiology and the availability of new therapeutics, MM is still considered an incurable disease (Anderson et al., 2002; Chatterjee et al., 2006; Tassone et al., 2006; Podar et al., 2009; Raab et al., 2009).

All animal models have benefits and limitations, and a single model cannot reproduce all pathophysiology. To select appropriate models for the study, we should consider many factors; an intact or deficient host immune system, the extent and severity of induced bone lesions, injection of human cell lines or primary human myeloma cells, similarity of the host microenvironment to human, and so on (Lwin et al., 2016). This review will focus on the highlights of these in widely used MM models on the aspect of MMBD.

### 5TMM-Derived C57BL/KaLwRij Models

The 5T mouse model of myeloma was initially reported by Radl et al. (1979), the most well-characterized syngeneic model. The model was established by serial transplantation of the BM of tumor-bearing mice into young syngeneic recipient C57BL/KaLwRij mice (Radl et al., 1988). It resembles features of the human benign MGUS stage (Radl, 1981). From this model, several mouse myeloma cell lines were established: 5T2MM, 5T33MM, and 5TGM1 (Radl et al., 1979; Radl et al., 1988; Manning et al., 1992; Asosingh et al., 2000).

The 5T2MM cells originate from spontaneously developed myeloma in elderly C57BL/KaLwRij mice (Radl et al., 1979; Radl et al., 1988). The model was generated by intravenously injecting BM cells from spontaneously developed MM mice into young syngeneic mice (Vanderkerken et al., 1999). This model can represent early human disease as it has moderate cell growth exhibiting slow tumor progression, which takes approximately 12–13 weeks post-transplantation. It comprises osteolytic lesions and trabecular bone loss from a reduction in OB number and an increase in OC number (Heath et al., 2009). It was the first animal model to evaluate a bisphosphonate effect on bone lesions (Radl et al., 1985). Zoledronic acid (Zol) is a potent anticatabolic nitrogen-containing bisphosphonate prescribed to high bone turnover patients. Croucher et al. (2003) demonstrated that Zol significantly reduces OC perimeter and bone lesions and increases trabecular bone and bone mineral density (BMD) in these mice compared to controls. Zol reduced a tumor burden and increased mouse survival (47 days from 35 days). Osteoprotegerin (OPG, a known inhibitor of osteoclastic bone resorption) was tested in the 5T2MM model as a therapeutic agent to reduce osteolytic bone disease in MM by inhibiting the interactions of the receptor activator of nuclear factor κB ligand (RANK) and its receptor (RANKL). Recombinant OPG was injected into a 5T2MM-bearing mouse that results in a significant reduction of bone lesions and OC and prevention of trabecular bone loss and increases of BMD and cortical bone thickness (Croucher et al., 2001). The dickkopf 1 (DKK1) has been shown to promote tumor cell–induced bone disease via inhibition of Wnt signaling. Furthermore, anti-DKK1 antibody treatment reduces trabecular and cortical bone loss and osteolytic lesions and increases the OB numbers and perimeter in 5T2MM-bearing mice (Heath et al., 2009). These collected results showed the use of 5T2MM model to explore MM development or bone disease–associated molecules, although the 5T2MM cells cannot be maintained in *in vitro* system as they are dependent on the BME.

Other 5T sub-cell lines have also been used in C57BL/KaLwRij mice to mimic human disease. 5T33MM cell line is another well-characterized model. This model represents an “aggressive” MM model due to rapid tumor growth (Asosingh et al., 2000). This model resembles advanced or relapsed MM phenotypes compared to the 5T2MM model. 5T33MM cells are no longer dependent on the BM environment and could grow in the *in vitro* system (Asosingh et al., 2000). Several studies had evaluated the therapeutic benefits of chemotherapies and cytotoxic agents in this model to target tumor burden (Khong et al., 2008; Xu et al., 2012). However, the 5T33MM model did not exhibit bone disease following tumor transplantation but showed destruction of cortical bone and reduced trabeculae at invaded sites (Vanderkerken et al., 1997). Subsequent studies showed no evident osteolytic lesions in the 5T33MM model (Buckle et al., 2012; Deshet-Unger et al., 2016).

The 5TGM1 model is a subsequent subclone of the 5T33 line. It was established via serial *in vivo* passage of the 5T33MM cells, used to investigate the mechanisms underlying MM-induced bone disease, as well as tumor burden (Garrett et al., 1997). Because of its short latency period, the 5TGM1 model can be used to test drugs to target both tumor burden and bone disease, which makes this model a cost-effective and reproducible model of human MM disease. The main advantage of the 5TGM1 model is its “pronounced” osteolytic lesion formation (Asosingh et al., 2000). Several studies have been made to target MMBDs in the 5TGM1 model. One study showed the effect of bisphosphonate ibandronate (IB) upon bone disease in the 5TGM1 model. 5TGM1-bearing mice treated with IB showed a reduction in osteolytic lesions in their vertebrae and long bones (Dallas et al., 1999). It also showed that IB also prevented trabecular and cortical bone loss. Systemic injection of 5TGM1-GFP cells into C57BL/KaLwRij mouse allowed tracing of individual myeloma cells and showed that they colonize the endothelial niche, entering a dormant stage. This dormant stage can be switched “on” by bone lining cells/OBs or “off” by OC. It may contribute to chemotherapy resistance, which targets divided cells (Lawson et al., 2015a). Furthermore, a recent study demonstrated that naive C57BL6/KaLwRij female mice injected with 5TGM1 producing small extracellular vesicle showed an increase in osteolysis, resulting in a significant reduction in trabecular bone volume without 5TGM1 cells. Furthermore, combination therapy of GW4869 (exosome secretion inhibitor) and BTZ in this model reduced tumor burden and bone lesions (Faict et al., 2018).

5T cell–bearing C57BL/KaLwRij model is the most studied animal model as it reproduces many features of human MM features, including MMBD. It has an intact immune system. Despite these benefits, it represents a single clonal murine MM-like disease, whereas human MM is highly heterogeneous.

### *MYC*-Derived MM Model

Another murine MM model, the Myc/Bcl-X_L_ mouse, was generated by the crossing hemizygous Myc transgenic mouse to hemizygous Bcl-X_L_ transgenic mouse (Cheung et al., 2004). It developed PC tumors in a relatively short period (average of 135 days) and 100% incidence. The mouse showed malignant PC infiltration into the BM, production of monoclonal immunoglobulin, and various osteolytic lesions (Cheung et al., 2004). This transgenic model is extensively used as it entails overexpression of recognized MM oncogenes under the control of an immunoglobulin enhancer.

Constitutive expression of a potent oncogene, *Myc*, in early B cells caused extramedullary lymphoma in Vk-Myc mouse. Vk-Myc mouse was developed under the control of mouse immunoglobulin κ (Igκ) light-chain gene-regulatory elements (Robbiani et al., 2005). Thereafter, Chesi et al. introduced early stop codon at *Myc* locus in Vk-Myc mouse to prevent its expression. Therefore, *Myc* can be sporadically activated in post-germinal B cells due to somatic hypermutation, resulting in the transition from the spontaneous MGUS to a disease that recapitulates the biological and clinical features of human MM. This model showed murine MM phenotype analogous to human MM disease with an indolent phenotype at the early stage of the disease and developed skeleton lesions compared to sex- and age-matched littermates (Chesi et al., 2008). A subsequent study performed a BM transplantation from Vk^∗^MYC mouse into sub-lethally irradiated congenic C57BL/6 mouse as a model representing relapsed refractory MM (Chesi et al., 2012). Comparable to the 5T cells, two bortezomib-resistant myeloma cell lines, Vk12653 and Vk12598, were established from aged Vk^∗^MYC mouse and develop myeloma after engraftment into young syngeneic mice. The Vk12598 cell line completely responded to melphalan, while it showed partial response against doxorubicin treatment (Lwin et al., 2016). The VK^∗^MYC mouse with these cell lines demonstrated the *in vivo* exploration of single and combinatorial therapy (Chesi et al., 2012). These models had been used to study the pathogenesis and the development of preclinical drugs, although skeletal evaluations were limited (Chesi et al., 2012; Matthews et al., 2013). Although it is out of this review’s scope, another beneficial feature of the Vk^∗^MYC model is imitating the accessible cross-talk among clonal PCs and nearby microenvironment. This characteristic allows for studying the clonal dynamics of the disease. A hypothesis of MM onset is based on the survival of competition among different clones at an early stage, which drives toward apathetic condition (Morgan et al., 2012).

The Vk^∗^MYC model emphasizes MYC’s multifaceted roles in MM commencement and growth and allows further modifications of MM progressor genes, transcription factors, adhesion molecules, and bone destruction mediators.

### *pE*μ*XBP-1* Transgenic Model

XBP-1, a transcription factor, is required for PC differentiation and is overexpressed in MM cells compared to normal PCs (Iwakoshi et al., 2003). The *E*μ*-xbp-1s* transgenic model was developed by the *Eu*-directed expression of the spliced *XBP-1* isoform (XBP-1s) (Carrasco et al., 2007). This mouse developed an MM-like disease by 280 days with changes in their skin, urinary cast, paraprotein production, splenomegaly, increased BM PCs, and shortened life span. The Eμ-xbp-1s mouse developed features such as elevated serum IgM and IgG, PC expansion ranging from 5 to 30% of total BM cellularity in the BM, and plasmacytic tumor development human MGUS or MM, and eventually lytic bone lesions. These features closely resemble MGUS and a significant proportion of MM progression.

Interestingly, this mouse’s disease appeared to advance with people from an MGUS-like state to an asymptomatic MM-like illness state by age. MM’s improvement’s long inertness period proposes that extra hereditary lesions are needed for full dangerous change. The transcriptional profiles of the lymphoid and MM cells show the liberation of qualities with known dysfunction in human MM, including cyclin D1, gp130, MAF, MAFB, CEBPs, BAFF, and APRIL (Carrasco et al., 2007).

### Human Myeloma Xenograft Model

Recombinase-Activating Gene-2 (RAG-2) deficient mice with impaired T and B cell development were employed to examine the BME (Fowler et al., 2009). Engraftment of 5TGM1 cells into RAG-2^–/–^ mouse resulted in myeloma development, associated with tumor growth within the bone and osteolytic bone disease and other features similar to humans. This study generated a double deficient (RAG2^–/–^MMP-9^–/–^) mouse and demonstrated significant reductions of myeloma cells in the BM and OC number in the 5TGM1 bearing RAG2^–/–^MMP-9^–/–^ mouse (Fowler et al., 2009). Postnov et al. (2009) generated two human MM cells, U266 and RPMI-8226/S, with the *luciferase*-*GFP* genes and engrafted these cells into total-body irradiated RAG2^–/–^γc^–/–^ mice at 24 h before transplantation. It showed that MM cells spread across the skeleton and severe bone lesions (Postnov et al., 2009).

A study demonstrated that the human immunoglobulin was produced in severe combined immunodeficient (SCID) mice engrafted with human peripheral blood mononuclear cells (Cavacini et al., 1992). It reduced the concern about the inconsistency of the host/donor associated features by human cell engraftment into mice and expanded a xenograft study. Many xenograft MM models are generated using various immune-compromised mice, including SCID, non-obese diabetic (NOD)/SCID, NOD.Cg-*Prkdc^*scid*^Il2rg^TM 1S*ug*^*/ShiJic (NOG), and NOD.Cg-*Prkdc^*scid*^Il2rg^TM 1W*jl*^*/SzJ mouse (NSG). In the absence of a mouse immune system, these xenograft models have been used to develop MM via systemic injection or orthotopic injection with human MM cell lines or patient BM/MM cells. These models offer the opportunity to evaluate human MM cells in the BME and test therapeutics’ effectiveness against human myeloma *in vivo*. But these models have limitations that do not reproduce complete tumor environments. A number of studies exhibited their abilities to reproduce many key features of human MM. Many studies have tested anti-tumor and bone-modulating drugs to target MM *in vivo*, and some have identified key molecules of bone destruction.

#### SCID and NOD/SCID Mouse

A variety of different myeloma cell lines including RPMI-8226 (Mitsiades et al., 2003; Wu et al., 2007; Thirukkumaran et al., 2012; Kikuchi et al., 2013), U266 (Mirandola et al., 2011; Kikuchi et al., 2013), KMS-11 (Carlo-Stella et al., 2006), KMS-12- BM (Rabin et al., 2007), and MM.1S (Wu et al., 2007; Azab et al., 2014; Roccaro et al., 2014; Swami et al., 2014; Zhang et al., 2014), as well as primary patient-derived myeloma cells (Pilarski et al., 2000), have been transplanted into SCID and NOD/SCID mice.

Human GFP expressing RPMI-8226/S MM cells was intravenously injected into SCID/NOD mice (Mitsiades et al., 2003). Whole-body constant fluorescence imaging was conducted to identify the location of GFP^+^ MM cells. Their anatomical dispersion and pathophysiological indications were agreed to the clinical course of MM in human patients, i.e., the pivotal skeleton (e.g., spine, skull, and pelvis) damage and advancement of loss of motion auxiliary to spinal lesions. This work first described the diffuse bone infiltration of human MM and a sequential bone disease progression. It subsequently provided a significant *in vivo* framework for the axial skeleton study.

Human myeloma cell line, U266, was intravenously injected into NOG mice, and all of these animals developed hindlimb paralysis after transplantation. Conversely, U266 cells in SCID or NOD/SCID mice did not show any myeloma features. In these mice, osteolytic injuries in cortical bones and loss of trabecular bones were found (Miyakawa et al., 2004). It demonstrated that this hu-myeloma NOG model might help study the pathogenesis of myeloma and related osteolytic lesions. NOD/SCID mice were intratibially injected with luciferase-expressing U266 cells and evaluated bortezomib, melphalan, aminopeptidase inhibitor, and histone deacetylase inhibitor for its applicability on preclinical drug tests (Fryer et al., 2013). Treatment with these drugs reduced the augmentation in all disease markers in mice. In addition, this model demonstrated successful myeloma development with primary myeloma cells from PC leukemia.

NOD/SCID animals with the luciferase-expressing myeloma cell (Luc^+^ MM1S) injection were treated with bone-targeting nanoparticles and evaluated MM progression and MM-induced bone lesions (Swami et al., 2014). This model demonstrated the tremendous potential of the bone-targeted drugs in the BME. The study suggests that NOD/SCID models can adapt interactions between MM cell and BME.

The OPG role was studied in co-transplantation of OPG overexpressing mesenchymal stem cells (MSCs) and KMS-12-BM cell into β2m NOD/SCID mouse (Rabin et al., 2007). This study demonstrated that OPG expressing MSCs caused the reduction in trabecular bone loss in the vertebrae and tibiae of myeloma developed animals.

A SCID mouse with intratibially transplanted 5TGM1 cells developed all myeloma features (Mori et al., 2004). BM stromal cells (BMSCs) from the 5TGM1-injected tibias and 11 MMBD (stage 3) patients express high levels of transcription repressor growth factor independence-1 (Gfi1) to suppress Runx2 via epigenetic changes in the Runx2 promoter (D’Souza et al., 2011). This report demonstrated that Gfi1 is a transcriptional repressor of the OB transcription factor Runx2 under osteogenic conditions, thus preventing osteoblastogenesis in MMBD.

#### Human Xenograft in NSG Mouse

A variety of myeloma cell lines, JJN3 (Lawson et al., 2015b), OPM2 (Fuhler et al., 2012; Lawson et al., 2015b), U266 (Dewan et al., 2004; Miyakawa et al., 2004; Bartee et al., 2012a; Bartee et al., 2012b; Lawson et al., 2015b), RPMI-8226-Luc (Hurchla et al., 2013), MM.1S (Bartee et al., 2012a), HuNS1 (Bartee et al., 2012a), L363 (Udi et al., 2013), and KMM-1 (Dewan et al., 2004), as well as primary patient-derived MCs (Lawson et al., 2015b), have been administered to NSG mice, which results in classical features of MM including paraplegia, paraprotein in the serum, osteolytic lesions, and loss of trabecular bone. A study performed intravenous injection of JJN3, OPM2, and U266 cell lines into NSG mice and demonstrated steady improvements of osteolytic bone lesions without dissemination outside of the BM in these models. Bone loss has been observed after Zol or Carfilzomib treatment with the RPMI-8226 cell engraftment. This study showed that Zol treatment reduced bone damage and tumor burden, whereas Carfilzomib reduced tumor burden alone (Lawson et al., 2015b). Another study showed that the Oprozomib, an oral proteasome inhibitor, reduced tumor burden, tibial trabecular bone loss, decreased serum c-terminal telopeptide (a bone resorption marker), and increased procollagen I N-terminal propeptide (a bone formation marker) levels in RPMI-8226-Luc–bearing NSG mice (Hurchla et al., 2013). The Osteolytica is a newly developed image analysis software to analyze three-dimensional (3D) cancer-induced osteolytic lesions in mice and evaluated U266-bearing NSG model (Evans et al., 2016). It showed that NSG mice are reliable *in vivo* model for improved clinical representation. In U266-GFP-Luc cell–transplanted NSG mice, MMBD was serially evaluated using *in vivo* micro–computed tomography (microCT), and it showed the antiresorptive capacity of transforming growth factor β (TGF-β) receptor I kinase inhibitor (SD-208) by prevention and treatment of myeloma bone lesions via enhancing collagen matrix maturation (Green et al., 2019). A combination treatment of anti–TGF-β–neutralizing antibody (1D11) and Zol was tested in the JJN3 and U266 intravenously transplanted NSG mice using *ex vivo* microCT and *in vivo* bone assessment at right tibias (Paton-Hough et al., 2019). In both models, the combination therapy showed the prevention of myeloma-induced bone lesions and bone formation from established bone lesions. This study suggests the combined antiresorptive and bone anabolic therapy as a new regimen for the patient. This study examined only tibias of the treated mice. NSG mouse is a potential model to demonstrate the role of cell adhesion molecules such as CD166 in MM progression. CD166 is highly expressed in various MM cell lines and MM patients’ primary BM cells. H929-GFP cells with CD166 were systemic and orthotopically injected into irradiated NSG mice. These models showed that CD166 affects MM progression by manipulating bone remodeling and decreasing trabecular thickness and bone volume, leading to bone lesions (Xu et al., 2016).

The main disadvantage of these models is the use of tumor cell lines. Most of the myeloma cell lines had been derived from the extramedullary MM stage or undergone aggressive transformations. They may not represent the typical apathetic stage of MM, although there were several attempts to use the primary MM cells to evade such limitations. These primary cells were also from relatively late stages, probably due to the low take rate of early-stage primary MM cells.

### SCID-hu MM Model

The SCID-hu model provides a human microenvironment to grow myeloma cells in the mouse and allow primary myeloma cells to grow. Human myeloma cells were inoculated into the human fetal bone chip implanted into irradiated SCID mice in this model (McCune et al., 1988). The advantage of this model is that human myeloma cells will grow in a human BME rather than a mouse.

Urashima et al. (1997) first showed engraftment and proliferation of human MM cell lines (ARH-77, OCI-My5, U-266, or RPMI-8226) in BM cavity of human fetal bone chips implanted into irradiated SCID mice and the MM cells homing exclusively to the human BM, but not in the murine BM. Afterward, Yaccoby et al. demonstrated that engrafted purified primary cells from MM patients in SCID-hu allow expansion and the development of several MM manifestations, including bone lesions, human paraprotein, and high blood calcium levels. Newly formed blood vessels were found in areas infiltrated by MM cells, demonstrating active angiogenesis (Yaccoby et al., 1998). Another study from the same group reported that inhibitor treatments for OC or the RANKL on the primary myeloma cells bearing SCID-hu reduced bone resorption and tumor burden, but such treatments to the mice with primary myeloma cells from the extramedullary disease patient did not affect this (Yaccoby et al., 2002). The SCID-hu model showed engrafting primary myeloma cells from medullary MM cells with slower growth rates and BM dependency (Li et al., 2007). Although these MM cells were hyperdiploid and grew strictly in the BM, these myeloma cells were originated from high-risk myeloma. Although the SCID-hu model with primary MM cells is biologically relevant to study MM pathophysiology, a limited mouse number (2–4 mice) produced from each patient sample is hard to test any therapeutic drug. Inoculation of INA-6 cells [interleukin 6 (IL-6)–dependent human MM cell line] into SCID-hu mice resulted in tumor engraftment and burden in the implanted bone with an increase in soluble human IL-6 receptor (shuIL6R) and human IL-6 in this mouse (Tassone et al., 2005). Although skeletal effects were not evaluated, many studies were performed in the INA-6 SCID-hu model for the anti-MM activity of the anti-inflammatory drug atiprimod (Neri et al., 2007), the anti-DKK1 monoclonal antibody BHQ880 (Fulciniti et al., 2009), the IκB kinase inhibitor, β-MLN120B (Hideshima et al., 2006), and a telomerase inhibitor (Shammas et al., 2008).

Although the SCID-hu MM model remains a consistent model to review the human disease and offers an important preclinical model, some boundaries remained: the allogeneic nature of the fetal BM environment versus MM cells and the genetic heterogeneity of implanted bone chips of human with patient MM cells collected from different individuals at dissimilar ages.

### SCID-rab MM Model

The SCID-rab model employs a rabbit bone subcutaneously implanted into SCID mice to avoid human fetal bone use (Yata and Yaccoby, 2004). The successful engraftment of BM cells from MM patients or CD138^+^ PCs into the SCID-rab reproduced matching M-protein isotype production and other myeloma clinical signs, including severe bone resorption of the implanted rabbit bone. The myeloma cells grew exclusively in the rabbit bone, whereas the myeloma cells from extramedullary disease patients grew on the outer surface of the implanted rabbit bone (Yata and Yaccoby, 2004). The authors claimed that the SCID-rab model presents a consistent and efficient model. As a rabbit is phylogenetically close to primate than a rodent, rabbit bone use was scientifically reasonable. The microenvironment of a rabbit bone can maintain the sustained primary human MM cell growth from most of the patients they tried.

For the study associated with MMBD, an important weakness of the model is the evident species difference between the rabbit bone and the human adult bone and the possible inconsistency of interactions of human MM cells with rabbit bone.

### SCID-Synth-hu Model

The SCID-synth-hu was developed to conquer the inadequacy of the SCID-hu system (Calimeri et al., 2011; DeWeerdt, 2011). This model is based on implanting a 3D bone-like poly-caprolactone polymeric scaffold into an SCID mouse. This model reproduces the microarchitecture of a normal human femur adult bone and permits the efficient coating of the 3D scaffold internal surface with human BM stem cells that produce the environment that effectively support the human primary MM cells engraft/growth. It should be noted that the allogeneic nature of the settings overcomes limitations associated with the SCID-hu model. The model achieved primary MM cells engraftment within the microenvironment that characterizes a significant development in the accessibility of prevailing *in vivo* environments. Preclinical evaluations of anti-MM drugs demonstrated considerable inhibition of the MM growth *in vivo*. Because of a lack of natural bone, bone absorption was not studied in this model.

### Pathophysiology and Etiology of MMBD

Bone disease is a significant component of MM. Many factors have been reported in the pathogenesis of MMBD. These factors play roles in tumor development and endurance. The association of PCs with BMSCs in the BME is critical for OC’s activation and proliferation (Terpos et al., 2003). OC stimulating factors were first described in 1974 (Mundy et al., 1974). Thereafter, many factors have been reported to be involved in the pathogenesis of MMBD. Although IL-1β and tumor necrosis factor α (TNF-α) are potent cytokines observed in MM patients, the IL-1β protein was not detected in clonal PCs from patients with both MM and MGUS, whereas TNF-α protein and mRNA were detected. Sati et al. (1999) concluded that myeloma cells produce TNF-α but not IL-1β. But a later study showed that elevated IL-1β mRNA levels were positively associated with bone lesions in patient biopsies (Donovan et al., 2002). Further research for the role of elevated IL-1β transcripts may be necessary.

BMSC secretes IL-6 after their interaction with myeloma cells. Also, IL-6 is an intense OC-stimulating factor for OC progenitor and induces bone resorption via OC (Reddy et al., 1994). Its essential impact on OC is an expansion of the early OC pool. However, studies have shown that TGF-β1 is also produced by BMSCs and can regulate IL-6 secretion by several tissues, including BMSCs. Furthermore, anti–TGF-β1 monoclonal antibody treatment blocked IL-6 secretion by BMSCs and also inhibited the increments in IL-6 secretion by BMSCs induced by MM cell adhesion (Urashima et al., 1996), suggesting the role of TGF-β1 in tumor cell proliferation.

TNF is found in the supernatant of PC cultures from MM patients, and hepatocyte growth factor (HGF) makes OC-like cells secrete IL-11. As TGF-β1 and IL-1 overload the impact of HGF on IL-11 production, it was postulated that HGF from myeloma cells induces MMBD via IL-11 (Hjertner et al., 1999). Later, elevated serum HGF levels were found in MM patients (Seidel et al., 2002).

Many studies reported that myeloma cells secrete factors that influence OB, OC, or both. The PC analyses from the patients with myeloma and control subjects revealed unique factors related to osteolytic lesions (Tian et al., 2003). This seminal work narrowed down to four genes significantly overexpressed in the patient PCs with magnetic resonance imaging–detected focal lesions. One of these genes, DKK1, was further investigated because of its known OB function. Immunohistochemical examination on BM biopsy revealed that only myeloma cells contained discernible DKK1. High DKK1 levels in BM plasma and patient blood were correlated to DKK1 expression and associated with the focal bone lesions. Recombinant human DKK1 or high DKK1-containing serum restrained OB differentiation in *in vitro* test.

High levels of macrophage inhibitory protein-1α (MIP-1α) were found in marrow samples from patients with MM but not in marrow from patients with other hematologic disorders or controls (Choi et al., 2000). Treatment of neutralizing antibody to MIP-1α to human BM cells with MM patients’ marrow plasma prohibited OC differentiation. These results support that MIP-1α plays an important role as the major factors responsible for the increased OC formation in patients with active MM. Furthermore, human MIP-1α enhanced OC formation from OC progenitors in combination with IL-6, parathyroid hormone–related protein, and RANKL at the later stages of OC differentiation (Han et al., 2001).

Vascular endothelial growth factor (VEGF) and its receptor, VEGFR, is upregulated in myeloma BM. VEGF induces vascularization and promotes angiogenesis with the progression of MGUS, leading to myeloma development (Ria et al., 2020). It has been reported that interaction of VEGF secreted by MM cells with OC-producing osteopontin not only increases vascular tubule formation but also induces osteoclastogenic activity by vascular endothelial cells. Thus, the interactions of myeloma cell, OCs, and vascular endothelial cells play a critical role in bone lesions, angiogenesis, and myeloma development (Tanaka et al., 2007). Further study revealed that VEGF binds to its receptor in the OCs and directly increases bone resorption and survival of mature OCs. There is also a positive feedback loop between VEGF expression by MM cells and IL-6 production from stromal cells (Voskaridou and Terpos, 2004).

Understanding the role of the RANKL/RANK/OPG system in bone remodeling provided a new perspective to pathophysiological studies of MMBD. RANK, RANKL, and the decoy receptor, OPG, are responsible for the regulation of OC production and activity. RANKL expressed in T lymphocytes, OBs, osteocytes, and bone stroma cells interacts with RANK secreted by OC precursor cells. The RANK–RANKL axis plays an important role in bone resorption (Voskaridou and Terpos, 2004; Boyce and Xing, 2008; Walsh and Choi, 2014). OPG, a blocker of RANKL-RANK interaction, inhibits activation and differentiation of OC. In fact, bone resorption can be dependent on the balance between RANKL and OPG expression. Studies have shown that RANKL expression in preosteoblastic or stromal cells in the coculture system is increased by human myeloma cells at both mRNA and protein levels, whereas OPG is downregulated (Voskaridou and Terpos, 2004). Studies have shown that serum RANKL/OPG ratios can be considered a potential prognostic marker in MM patients. A greater ratio represents the formation and activation of OCs, leading to shorter survival. Moreover, soluble RANKL level is associated with the extent of bone disease (Sezer et al., 2003; Raje et al., 2019).

Sclerostin produced by osteocytes binds to Wnt coreceptor, LRP5/6, and inhibits the canonical Wnt pathway (Delgado-Calle et al., 2017). MM patients have high serum sclerostin, whereas MGUS and smoldering MM patients have only low levels (Eda et al., 2016). Sclerostin serum level is positively correlated with reduced OB activity and poor survival (Terpos et al., 2012). The osteocytes in JJN3 intratibially injected mice expressed high sclerostin levels and significant bone lesions (Delgado-Calle et al., 2016). In addition, the coculture of osteocytes with myeloma cells showed substantial reductions of OPG and OB differentiation markers. These data provided evidence of interactions among Wnt signaling, OB differentiation, and sclerostin. Besides, coculture of OBs from MM patients with INA-6 or H929 MM cells showed that MM cells inhibit OB differentiation via sclerostin from DKK-1–stimulated premature OBs (Eda et al., 2016). Treatment with antisclerostin increased femur trabecular bone volumes in 5TGM1-bearing or naive C57BL/KaLwRij mice and suppressed MM1S tumor progression in SCID (Reagan et al., 2015). A subsequent study demonstrated that antisclerostin treatment prevented myeloma-induced inhibition of bone formation, but did not affect osteoclastic resorption or tumor burden. A combined treatment with antisclerostin and Zol showed additive effects (McDonald et al., 2017).

Studying the pathophysiology of MMBD provides a better understanding of underlying mechanisms responsible for bone remodeling imbalance, leading to proper design and selection of animal models for the study. These efforts resulted in developing targeted drugs not only for MMBD but also for other bone metastasis disease. The RANKL rivals, OPG-Fc and RANKL-Fc, showed inhibition of the RANKL activity (Schwarz and Ritchlin, 2007). As AMG162, a human monoclonal antibody against RANKL, demonstrated compelling data in diminishing bone turnover markers in postmenopausal women (Bekker et al., 2004), AMG162 has been under scrutiny in clinical preliminaries. Denosumab, AMG162, was a well-tolerated drug and reduced significant and prolonged reduction of bone resorption in MM, as well as bone metastases from breast cancer (Body et al., 2006). A recombinant OPG, AMGN-0007, was developed as a potential therapeutic agent in bone disease. A single dose of AMGN-0007 showed significant decreases of urinary N-telopeptide of collagen/creatine in MM patients (Body et al., 2003).

## Conclusion

Animal models allow specific hypothesis-driven researches and enable researchers to address specific questions. Syngeneic models of the tumor are the models in which the tumor cell line is derived from the same species as a recipient. Therefore, it is easy to test the efficacy of immuno-oncology agents as single therapies or in combination with other anticancer drugs. The syngeneic model has several advantages that include the use of mice with an intact immune system. Besides, these models demonstrate high tumor cell engraftment rates and low variability. These are beneficial to study the efficacy of novel antitumor agents. Many MM models have been developed and allowed to interrogate the mechanism of MM tumorigenesis in murine backgrounds. But these models represent a single clonal MM-like disease in the murine system, whereas human MM is a highly heterogeneous disease. Such limitation leaves gaps of understanding of human MM and limits of translation to the clinic.

Employing immunodeficient mice with human MM cell transplantation was tried to human MM cells and reproduced many human MM features. Human MM cell lines and even patient MM cells used in these models represent the aggressive MM stage as transplanted myeloma cells were transformed during passages and/or obtained from the late stages of MM. Besides, a discrepancy of human myeloma cells from mouse BME leads to limit its reproducibility. Xenograft models are functional tools for the preliminary selection of novel drugs predominantly in the absence of pharmacological information, but they are not considered as perfect models for the development of anti-MM drugs. Indeed, the concerns on the non-human host stroma have been increased in relation to the pathophysiological condition in patients. Therefore, mouse xenograft models generally serve as a useful tool for studying the significant antitumor activity and a potent anti-MM drug’s pharmacodynamic assets.

The models from the engraftment of human primary MM cells in a human BME can prevail over the weaknesses of conventional xenografts or genetically modified mice. The SCID-hu models are useful to investigate the disease pathophysiology and evaluate new drugs that target the tumor and the human BME.

We summarized the animal models with their strengths and limitations in the summary Table 1. While we review, we noticed that many studies were done in the femur, tibia, or implanted bone chips rather than the spine, one of the sites frequently found at the clinic and significantly impacting patient’s life quality. The femur or tibia was chosen because it has a relatively simple structure and is accessible to an orthotopic injection. Besides, reports showed relatively mild bone damage at the spine than tibia due to systemic infusion of myeloma cells. The MMBD at the spine may not be the same as the ones at the femur or tibia. It will be worth investigating in future studies.

**TABLE 1:** Classification of immunocompetent and immunocompromised animal models of MMBD with strengths and shortcomings.

**Model**	**Immune system**	**Category of model**	**Derived cells**	**Tested drugs**	**Strengths**	**Limitations**
5TMM derived models	Immuno-competent	Syngeneic	5T2MM (Radl et al., 1988)	Zoledronic acid (Croucher et al., 2003), osteoprotegerin (Croucher et al., 2001), and anti-DKK1 antibody (Heath et al., 2009)	a. Representative of early human MM disease (moderate cell growth and slow tumor progression) (Heath et al., 2009) b. To cause osteolytic lesions (trabecular bone loss) (Heath et al., 2009) c. To explore molecules associated with MMBD (Croucher et al., 2001, 2003; Heath et al., 2009) d. To study tumor burden and bone disease (Croucher et al., 2001)	5T2MM cells cannot be cultured *in vitro* (They are dependent on the BME) (Croucher et al., 2001; Heath et al., 2009)
			
			5T33MM (Manning et al., 1992)	–	a. Representative of advanced MM model (rapid tumor growth) (Asosingh et al., 2000) b. Cells can be cultured *in vitro* (not dependent on the BME) (Asosingh et al., 2000) c. To study tumor burden (Khong et al., 2008; Xu et al., 2012)	Do not exhibit bone disease (Vanderkerken et al., 1997; Buckle et al., 2012; Deshet-Unger et al., 2016)
			
			5TGM1 (Asosingh et al., 2000)	Ibandronate (Dallas et al., 1999; Lawson et al., 2015a), bortezomib, and GW4869 (Faict et al., 2018)	a. Cost-effective (short latency period) (Asosingh et al., 2000) b. Sever osteolytic lesion formation (Asosingh et al., 2000) c. To study tumor burden and bone disease (Garrett et al., 1997)	Resistant to chemotherapy when entering a dormant stage (Lawson et al., 2015a)

Vk*MYC	Immuno-competent	Transgenic	Vk12653 and Vk12598 (Lwin et al., 2016)	Bortezomib, melphalan, and doxorubicin (Lwin et al., 2016)	a. Representative of murine MM phenotype analogous to human MM disease (Chesi et al., 2008) b. Production of various osteolytic lesions (Chesi et al., 2008) c. Appropriate model to study pathogenesis, clonal dynamics of the disease and development of pre-clinical drugs (Chesi et al., 2012; Matthews et al., 2013)	Lack of true “MGUS” period in Vk*MYC model (Kuehl, 2008)

pEμXBP-1	Immune-deficient	Transgenic	–	–	To develop plasmacytic tumors resembling human MGUS or MM lytic bone lesions (Carrasco et al., 2007)	a. Shortened life-span (Carrasco et al., 2007) b. Additional genetic lesions are required for full malignant transformation (Carrasco et al., 2007)

RAG2^–/–^ MMP-9^–/–^	Immune-deficient	Xenograft	–	–	a. To examine the BME (Fowler et al., 2009) b. To develop tumor within the bone and osteolytic bone disease similar to human (Fowler et al., 2009) c. To examine the role of bone microenvironment due to the growth of myeloma cells in bone and non-bone sites (Fowler et al., 2009)	Not appropriate to investigate the immune system, specifically B and T cells, in myeloma (Fowler et al., 2009)

SCID, NOD/SCID, NOG, NSG	Immune-deficient	Xenograft	–	Osteoprotegerin (Rabin et al., 2007), zoledronic acid (Lawson et al., 2015b; Paton-Hough et al., 2019), 1D11 (Paton-Hough et al., 2019), carfilzomib (Lawson et al., 2015b), bortezomib (Fryer et al., 2013), melphalan (Fryer et al., 2013), and oprozomib (Hurchla et al., 2013)	a. To evaluate human MM cells in the BME (Mitsiades et al., 2003; Miyakawa et al., 2004; Mori et al., 2004; Lawson et al., 2015b) b. To test the effectiveness of antitumor and bone modulating drugs against human myeloma *in vivo* (Rabin et al., 2007; Fryer et al., 2013; Hurchla et al., 2013; Lawson et al., 2015b; Paton-Hough et al., 2019)	a. Do not reproduce complete tumor environments due to human/mouse differences b. Most of the derived myeloma cell lines used in these models do not represent the typical apathetic stage of MM
						
SCID-hu	Immune-deficient	Xenograft	–	–	a. Provides a human microenvironment to grow human myeloma cells in the mouse (McCune et al., 1988) b. Allow primary myeloma cells to grow (McCune et al., 1988) c. To study MM pathophysiology (bone lesions, human paraprotein, and high blood calcium levels) (Yaccoby et al., 1998) d. Associated with active angiogenesis (Yaccoby et al., 1998)	a. Engrafting primary myeloma cells with slow growth rate b. Limited mouse number (two to four mice) is produced from each patient sample
SCID-rab	Immune-deficient	Xenograft	–	–	a. Rabbit bone microenvironment is more similar to human bone microenvironment than a rodent (Li et al., 2007) b. Exclusive growth of the myeloma cells in the rabbit bone (Li et al., 2007)	Possible inconsistency of interactions of human MM cells with rabbit bone

SCID-synth- hu	Immune-deficient	Xenograft	–	–	a. To reproduce microarchitecture of a normal human femur adult bone to support the human primary MM cells engraft/growth (Calimeri et al., 2011; DeWeerdt, 2011) b. To evaluate anti-MM drugs (Calimeri et al., 2011; DeWeerdt, 2011)	Inappropriate to study bone absorption (Lack of natural bone) (Calimeri et al., 2011; DeWeerdt, 2011)
						

Bisphosphonates are the only approved treatment for the prevention of MMBD. This anti-absorption therapy does not promote bone formation and cannot correct the established lesion. Current MMBD therapy is still aimed at pain control and prevention of skeletal fractures because of widespread belief that bone lesions do not heal. Recently, several clinical observations revealed that myeloma-induced bone lesions could be recovered (Hinge et al., 2016; Mohan et al., 2017, 2021). Such observations raise a novel question—why some patients heal while others do not. As we previously described, bone anabolic drugs can potentially benefit patients who already have established bone lesions. We found that multidisciplinary efforts from preclinical and clinical sides have been made. Such efforts promise that new bone anabolic drugs will be available soon for MMBD patients.

At present, there is no perfect model for the study of MMBD. Therefore, a thorough understanding of each model’s advantages and disadvantages is necessary to choose an appropriate model to study or test the specific question(s).

## Author Contributions

SHM wrote the initial draft. SN revised and wrote several sections. SJM wrote a part of the section. CM and LM wrote sections of this article and edited the manuscript. DY planned, revised, and finalized this article. All authors contributed to the article and approved the submitted version.

## Conflict of Interest

The authors declare that the research was conducted in the absence of any commercial or financial relationships that could be construed as a potential conflict of interest.
